# ALG-2 couples T cell activation and apoptosis by regulating proteasome activity and influencing MCL1 stability

**DOI:** 10.1038/s41419-019-2199-4

**Published:** 2020-01-02

**Authors:** Tian-Sheng He, Wangsheng Ji, Junqi Zhang, Jing Lu, Xinqi Liu

**Affiliations:** State Key Laboratory of Medicinal Chemical Biology, College of Life Sciences, Nankai University, Tianjin, China

**Keywords:** Proteases, Apoptosis, T-cell receptor

## Abstract

T cell homeostasis is critical for the proper function of the immune system. Following the sharp expansion upon pathogen infection, most T cells die in order to keep balance in the immune system, a process which is controlled by death receptors during the early phase and Bcl-2 proteins in the later phase. It is still highly debated whether the apoptosis of T cells is determined from the beginning, upon activation, or determined later during the contraction. MCL1, a Bcl-2 family member, plays a pivotal role in T cell survival. As a fast turnover protein, MCL1 levels are tightly regulated by the 26S proteasome-controlled protein degradation process. In searching for regulatory factors involved in the actions of MCL1 during T cell apoptosis, we found that ALG-2 was critical for MCL1 stability, a process mediated by a direct interaction between ALG-2 and Rpn3, a key component of the 26S proteasome. As a critical calcium sensor, ALG-2 regulated the activity of the 26S proteasome upon increases to cytosolic calcium levels following T cell activation, this consequently influenced the stability of MCL1 and accelerated the T cell “death” process, leading to T cell contraction and restoration of immune homeostasis. Our study provides support for the notion that T cells are destined for apoptosis after activation, and echoes the previous study about the function of ALG-2 in T cell death.

## Introduction

The abundance of T lymphocytes in mammals is tightly regulated to ensure the proper function of the immune system. Both an excess and lack of immune cells can lead to the malfunction of the immune system, reflected as either an auto immune disease or immunodeficiency. Upon pathogen infection, antigen-specific T cells quickly proliferate by clonal expansion, resulting in a robust immune response against pathogen. Following the elimination of the pathogen, most of the T cells, except a small number with transition into memory cells, die through activation-induced cell death (AICD)^1,2^. The mechanism by which AICD is initiated still remains highly elusive. Some evidences suggest that AICD is determined from the beginning of T cell activation, and therefore T cell activation and T cell death are coupled processes. In contrast, some other studies show that T cell death is triggered during the contraction phase of T cells due to the alteration of environmental cues. The waning of the excessive T cells that have proliferated during the activated immune response is controlled by both death receptors and Bcl-2 family proteins^3,4^. Death receptors, such as the Fas, play a pivotal role in controlling the magnitude of immune response, while also enhancing cell death in the early stage of T cell contraction^5^. In the later stages of the immune attenuation, accompanying with the withdrawal of growth factors and activating cytokines, most of the cells die through a process controlled by the Bcl-2 family, which include both anti-and pro-apoptotic proteins, whose balance regulates the speed of T cell apoptosis^6^.

Within the Bcl-2 protein family, MCL1 (Induced myeloid leukemia cell differentiation protein) is unique due to its short half-life, rendering MCL1 very sensitive to the status of T cells. MCL1 protects cells from growth factor withdrawal, and is also critical in T and B cell development and survival^7–9^. Except for its C-terminal BH domains, which are conserved within the Bcl-2 family, MCL1 has a unique N-terminal extension including several critical phosphorylation and ubiquitination sites^10,11^. These sites render MCL1 sensitive to various survival cues from upstream kinases and ligases. MCL1 stability is tightly controlled by these upstream regulators, and the fast turnover rate helps MCL1 quickly respond to the environmental conditions surrounding the T cells. The ubiquitination of MCL1 by E3 ligases, such as MULE, leads to the degradation of MCL1 through the 26S proteasome-controlled ubiquitination-proteasome protein degradation pathway^12^. Therefore, the protein levels of MCL1 within cells are greatly affected by the activity of the proteasome. Reasonably, the degradation of MCL1 by the proteasome must be carefully regulated in accordance with the status of T cells, which defines the magnitude and the time course of adaptive immunity. Interestingly, the activity of the proteasome is tightly associated with T cell proliferation and differentiation. Following activation by infection, the activity of the proteasome is upregulated and the metabolism of the cell is changed from oxidative phosphorylation to oxidative glycolysis. Therefore, proteasome activity is affected by cellular metabolism and cell fate, while being regulated by various environmental cues.

The 26S proteasome is composed of a 20S core, and a 19S regulatory particle cap at either end^13^. The 19S regulatory particle includes a base (AAA-ATPase ring) and a lid subcomplex. The lid includes nine components (Rpns, 26S proteasome non-ATPase regulatory subunits), which are responsible for the assembly, recognition of substrate, and regulation of proteasome activity^14^. The activity of the proteasome is regulated by different regulators in a number of various physiological pathways. For example, activated PKA by raising cAMP can phosphorylate proteasome subunit Rpn6, leading to an increase to the hydrolysis activity of the proteasome^15^. Additionally, RAD6 promotes the activity of the proteasome by enhancing the degradation of PSMF1^16^. Another regulator of proteasome activity, Ecm26, inhibits proteasome assembly under oxidative stress^17^. Despite extensive investigation having been performed on proteasome regulation, few studies have been carried out in T cells, leaving us wondering if there are any new potential regulators of proteasome activity in T cells, which may play roles in MCL1 degradation and thereby influence T cell apoptosis. Taking this into consideration, we chose critical components of proteasome assembly, Rpn3, Rpn6, and Rpn11 as baits to search for the potential regulators of the proteasome within Jurkat T cells. In this search a new interacting partner of Rpn3, Apoptosis-linked gene 2 (ALG-2), also known as Programmed Cell Death 6 (PDCD6), was identified. Rpn3, also known as PSMD3, was a critical component of 19S regulatory particle. Rpn3 is known to be associated with the whole blood cell count and inflammatory diseases^18^. Additionally, it has been shown to be critical for the survival of white blood cells, especially neutrophils^19,20^. The proper functioning of Rpn3 is required for the degradation of cell cycle regulatory proteins, and is thus involved in cell cycle regulation^21^. Rpn3 also regulates insulin signal transduction, has roles in glucose-related dietary traits, and is involved in interferon-induced neutropenia during HCV infection^22,23^. Structurally, Rpn3 is suggested to localize to the surface of the lid subcomplex, with its C-terminal tail protruding into the ATPase ring, thereby regulating the entry of substrate peptides into the digesting core region^24^. Accordingly, the absence of Rpn3 results in the collapse of the proteasome. Therefore, Rpn3 is critical for the proper functioning of the proteasome and plays an important role in the immune response.

The identified interacting partner of Rpn3 in this study, ALG-2, has originally been identified in T cell apoptosis^25^. The anti-sense DNA of ALG-2 was found to rescue a T cell hybridoma from T cell receptor (TCR) and Fas-induced apoptosis, and thereby defined ALG-2 as a pro-apoptosis factor^25^. Intriguingly, follow-up studies shed doubt on this conclusion, since the *alg-2* knockout mice grow normally, as well as with functional T cell development and apoptosis, suggesting a redundancy, or non-critical function of ALG-2 in vivo. Even so, the significance of ALG-2 has been acknowledged, including its involvement in ESCRT-related vesicle transportation, cell plasma membrane repair, and inhibition of HIV infection^26–28^. Additionally, a number of ALG-2 interacting partners have been identified, including Alix^29,30^, TSG101^31^, HEBP2^28^, and SEC31^32–34^, which were found to interact with ALG-2 by either a type I (PPYPXXPGYP) or type II (PXPGF) ALG-2 binding motif^35,36^. ALG-2 is a calcium-binding protein with five EF-hand motifs, but only EF1 and EF3 have been identified to have strong calcium-binding ability^37^. The calcium-binding ability of ALG-2 is critical for its proper function. Conceivably, ALG-2 might function as a sensor for cytosolic calcium levels and initiate the signal for downstream proteins by a direct interaction. ALG-2 is ubiquitously expressed and its abnormal expression has been found in various cancers^38^. Therefore, ALG-2 might have a critical role in both cell development and survival, despite the existence of possibly redundant proteins.

This study showed that following T cell activation, ALG-2 enhanced the activity of the proteasome and promoted the degradation of MCL1 by a direct interaction with Rpn3, thus, coupling the T cell activation and apoptosis processes, shedding new light on the process of AICD. This study identified ALG-2 as a novel regulator of the proteasome and provided an explanation for its function in T cells.

## Results

### MCL1 levels are associated with serum starvation-induced T cell apoptosis

**Fig. 1:**
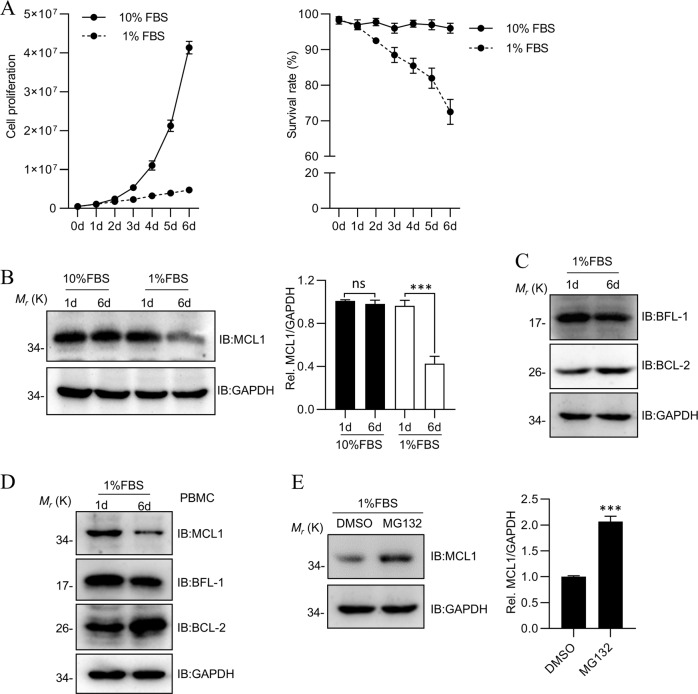
MCL1 levels are associated with serum starvation-induced T cell apoptosis. **a** The proliferation of Jurkat cells cultured in 10% FBS or 1% FBS medium. The assay was started with 500,000 cells and examined with Trypan blue staining using a Countstar cell-counter system. The experiments were repeated in three independent times. **b** The changes of MCL1 protein level in the 1% FBS culture medium. 1.5 × 10^6^ cells were collected on the fifth day and detected by MCL1 antibody. **c** The changes of BFL-1 and BCL-2 protein level in Jurkat cells on the sixth day cultured in the 1% FBS culture medium as **b**. **d**The changes of MCL1, BFL-1 and BCL-2 protein level in PBMCs cultured as **b**. **e** MCL1 level was restored partially by supply of MG132 in 1% FBS culture medium. MG132 was added into 1% FBS culture medium on the sixth day and collected for detection after 5 h. Data shown were representative of three independent experiments. Error bars indicate SD. ns, no significance; ***P < 0.001.

MCL1 has been shown to protect cells from growth factor withdrawal-induced cell death^7^. To explore the mechanism by which MCL1 is regulated in Jurkat T cells, we established a model of growth factor withdrawal by using 1% FBS to culture cells (Fig. 1a). MCL1 protein levels were found to be stable in nutrient-efficient proliferating cells (Fig. 1b), but dramatically decreased in cells subjected to serum starvation, which was accompanied by an increase to cell death (Fig. 1b). However, other BCL-2 family proteins, such as BCL-2 and BFL-1, showed mild differences in serum starvation (Fig. 1c). These results supported a critical role of MCL1 in T cell apoptosis triggered by growth factor withdrawal. Moreover, we repeated the experiment in peripheral blood mononuclear cells (PBMCs), and found MCL1 dramatically reduced in serum starvation (Fig. 1d). The MCL1 levels were partially restored with the proteasome inhibitor MG132, indicating that the proteasome-mediated degradation process played a major role in regulation of MCL1 protein levels (Fig. 1e).

### ALG-2 is a potential regulator of proteasome activity

**Fig. 2:**
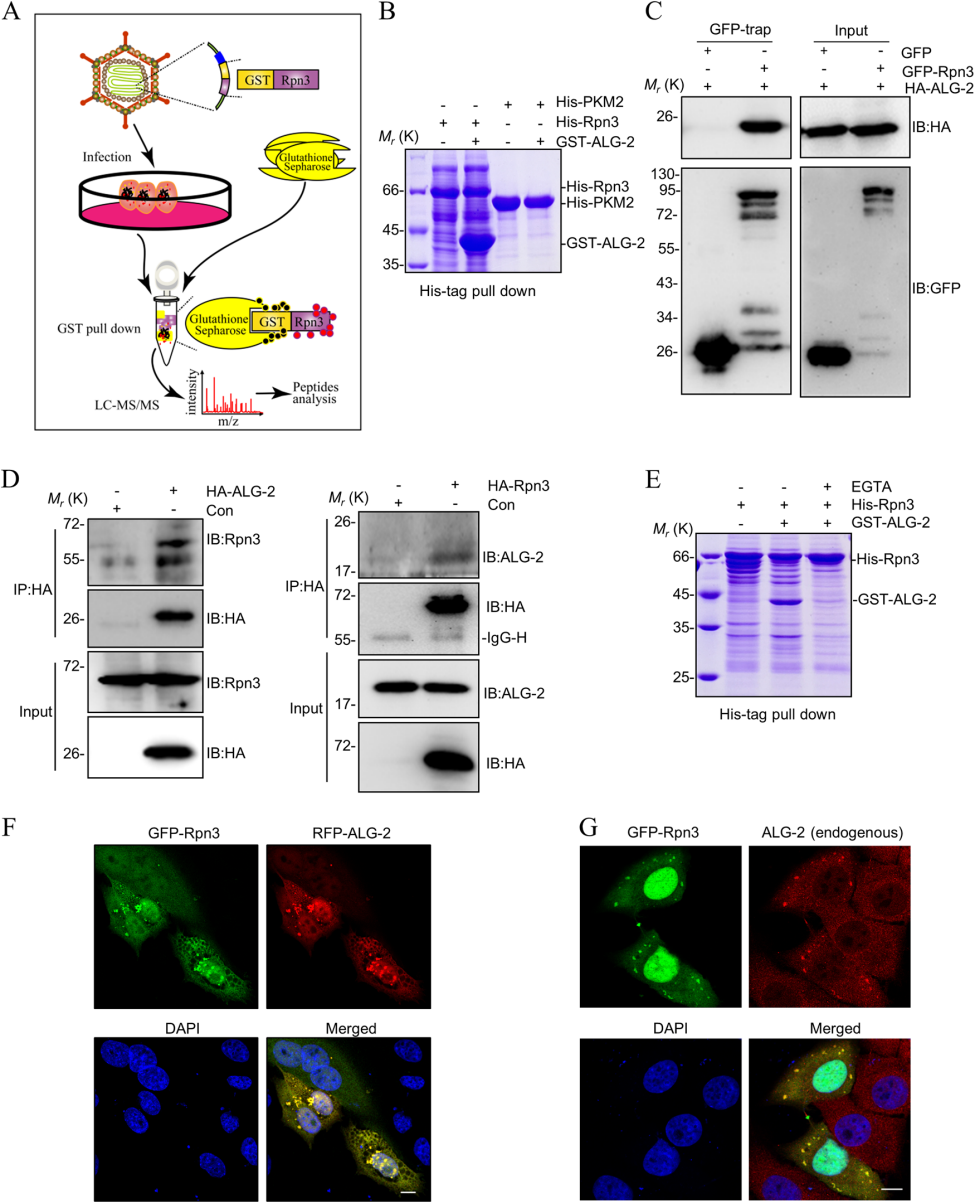
ALG-2 is a potential regulator of proteasome activity. **a** The sketch map to illustrate the assay of GST pull-down coupled with LC-MS/MS. Jurkat cells were infected with GST or GST-Rpn3 retrovirus for 72 h and lysed by NP-40 buffer. The samples were prepared by GST pull-down and analyzed by LC-MS/MS. **b** The interaction of Rpn3 with candidate protein ALG-2 was validated by His-tag pull-down in prokaryotic expression system. Purified proteins were detected by SDS-PAGE with coomassie blue staining. **c** ALG-2 interacts with Rpn3 in vivo. HEK293T cells were co-transfected with GFP-Rpn3 and HA-ALG-2 for 48 h and lysated by RIPA buffer. Cell lysates were immunoprecipitated with GFP-Trap sepharose beads and analyzed by western blot. **d** ALG-2 interacts with Rpn3 in endogenous expression levels. HEK293T cells were transfected with HA-Rpn3 or HA-ALG-2 for 48 h and lysated by RIPA buffer. Co-immunoprecipitation and immunoblot analysis were performed with the indicated antibodies. **e** ALG-2 and Rpn3 is dependent on the presence of Ca^2+^. **f**, **g** Colocalization of ALG-2 and Rpn3. HeLa cells were transfected with GFP-Rpn3 and RFP-ALG-2 (**f**) or GFP-Rpn3 only. Endogenous ALG-2 was analyzed by immunostaining with TRITC (**g**). Fluorescence was imaged by confocal laser scanning microscope. Scale bars: 10 μm.

The regulation of proteasome activity in T cells is not clearly characterized. To address this, a pull-down assay was performed using essential proteasome components, Rpn3, Rpn6, and Rpn11, as baits to search for the new binding partners in Jurkat T cells. Using this method, ALG-2 was identified as a new interacting protein of the proteasome by a direct interaction with Rpn3 (Fig. 2a and Table S1). The interaction between ALG-2 and Rpn3 was further confirmed by GST pull-down in prokaryotic expression system (Fig. 2b) and GFP-trap co-immunoprecipitation in eukaryotic cells (Fig. 2c). The further co-immunoprecipitation assays with endogenous proteins revealed that ALG-2 co-immunoprecipitated with endogenous Rpn3 (Fig. 2d left panel) and Rpn3 co-immunoprecipitated with endogenous ALG-2 (Fig. 2d right panel), and proved Rpn3 indeed interacts with ALG-2. Since ALG-2 has been reported to interact with multiple proteins (e.g. ALIX,SEC31A, MCOLN1) in a Ca^2+^-dependent manner^30,32,39^, we confirmed that the interaction between ALG-2 and Rpn3 is dependent on the presence of Ca^2+^ (Fig. 2e). Moreover, the co-expression of GFP-Rpn3 with either RFP-ALG-2 (Fig. 2f) or endogenous ALG-2 (Fig. 2g) showed co-localized of two proteins in granule or puncta in cytoplasm, which provided additional support for their direct interaction within cells.

### ALG-2 enhances the activity of the proteasome

Based on the interaction between ALG-2 and Rpn3, we speculated that ALG-2 may regulate directly the activity of the proteasome. To test this, we introduced a GFP-based substrate (Ub^G76V^-GFP), which could rapid quantification of ubiquitin/proteasome-dependent proteolysis in living cells^40^. The Ub^G76V^-GFP contained a mutant ubiquitin, which prevented cleavage of the N-terminal ubiquitin from the GFP and served as an anchor for polyubiquitination and degradation^40^. Consequently, Ub^G76V^-GFP was a good substrate for 26S proteasome-dependent proteolysis, and its fluorescence intensity was inversely proportional to proteasome activity^41^. As illustrated in Fig. S1A, ALG-2 induced a decrease of the fluorescence intensity of Ub^G76V^-GFP, but not GFP, in HeLa cells treated with cycloheximide (CHX), and the fluorescence intensity was increased with the addition of MG132.

**Fig. 3:**
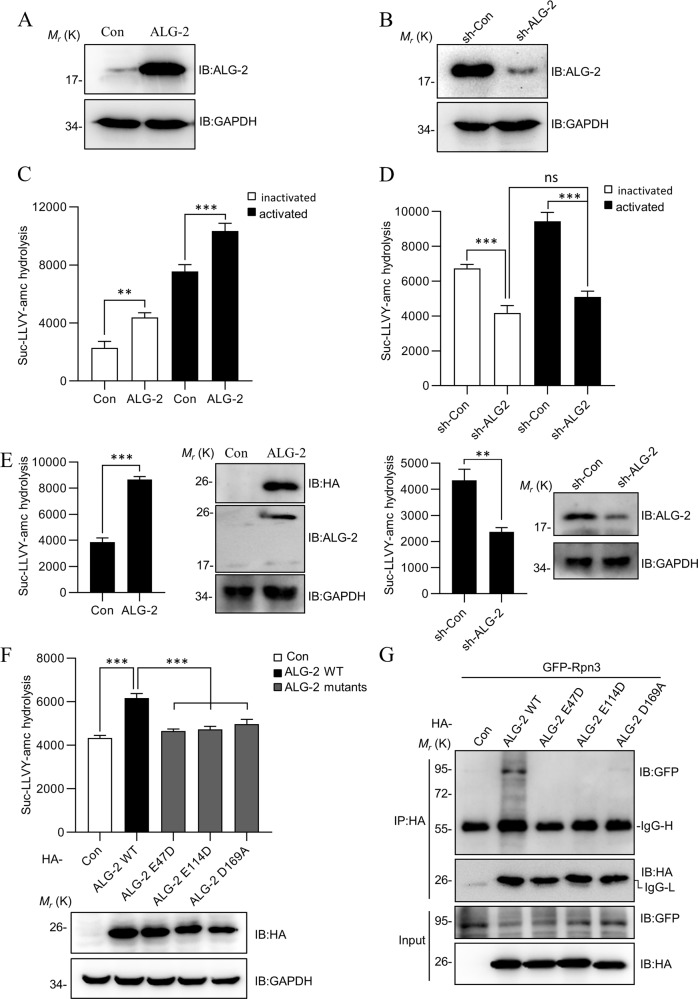
ALG-2 enhances the activity of the proteasome. **a**, **b** The expression levels of ALG-2 in ALG-2-OX and ALG-2-KD Jurkat cell lines. The protein levels of ALG-2 and GAPDH are determined by western blotting. **c**, **d** ALG-2 enhances the activity of proteasome in Jurkat cells. The ALG-2-OX and ALG-2-KD Jurkat cells, either inactivated or activated, were incubated in digitonin permeabilization buffer with slight sonication. Cell lysates were incubated for 1 h at 37 °C in reaction buffer with Suc-LLVY-AMC. The activity of proteasome was quantitated by AMC fluorescence intensity in a microtiter plate reader at *λ*^EX^ = 380 nm/*λ*^EM^ = 460 nm. **e** ALG-2 enhances the activity of proteasome in stimulated PBMCs. PBMCs were infected with ALG-2 retrovirus (left panel) or sh-ALG-2 retrovirus (right panel) for 40 h and activated by PMA and ionomycin for 10 h. Then the cells were measured the activity of proteasome as **c**. **f** ALG-2 enhances the activity of proteasome dependent on its calcium-binding sites. ALG-2-KD Jurkat cells were infected with ALG-2 WT, ALG-2 E47D, ALG-2 E114D, and ALG-2 D169A retrovirus for 48 h, then activated by PMA and ionomycin for 10 h. Proteasomal activities were assessed by Suc-LLVY-AMC cleavage as **c**. **g** Co-immunoprecipitation assays of ALG-2 mutants with Rpn3. Data shown were representative of three independent experiments. Error bars indicate SD. **P < 0.01; ***P < 0.001.

Next, we used a Suc-LLVY-AMC peptide to assesse the effect of ALG-2 on the chymotryptic activity in jurkat T cells. Both ALG-2 overexpressed (ALG-2-OX) and *alg-2* knockdown (ALG-2-KD) cell lines were established using a puromycin selection method (Fig. 3a, b). The overexpression of ALG-2 enhanced the proteasome activity, whereas knockdown reduced it (Fig. 3c, d). ALG-2 coordinated several calcium ions in a dimer form, and its calcium-binding ability was critical for its proper functioning. Thus, with the intracellular calcium level increasing dramatically following T cell activation, this may be sensed by ALG-2. To detect if the effect of ALG-2 on the activity of the proteasome was associated with the fluctuation of intracellular calcium levels, Jurkat T cells were activated by PMA (phorbol 12-myristate 13-acetate) and ionomycin. The overexpression of ALG-2 was found to increase the proteasome activity in both inactivated and activated T cells, whereas the knockdown of *alg-2* decreased it (Fig S1B and C, Fig. 3c, d). Furthermore, we determined the effects of ALG-2 on the proteasome activity in stimulated PBMCs, and found that ALG-2 consistently increased the proteasome activity (Fig. 3e). Interestingly, despite T cell activation by PMA/ionomycin enhanced the proteasome activity significantly in wild-type (WT) and ALG-2-OX cells (Fig. 3c), this effect was abolished in the context of *alg-2* knockdown (Fig. 3d). Meanwhile, mutations (E47D, E114D, and D169A)^42,43^ that abolished the calcium-binding ability of ALG-2 also lost the enhancement on the proteasome activity following T cell activation (Fig. 3f). The further co-immunoprecipitation assays revealed that ALG-2 mutants also lost interact with Rpn3 (Fig. 3g). This observation highlighted that the influence that ALG-2 had on the proteasome was dependent on the T cell activation status and the calcium level within the cells.

### ALG-2 accelerates MCL1 degradation

**Fig. 4:**
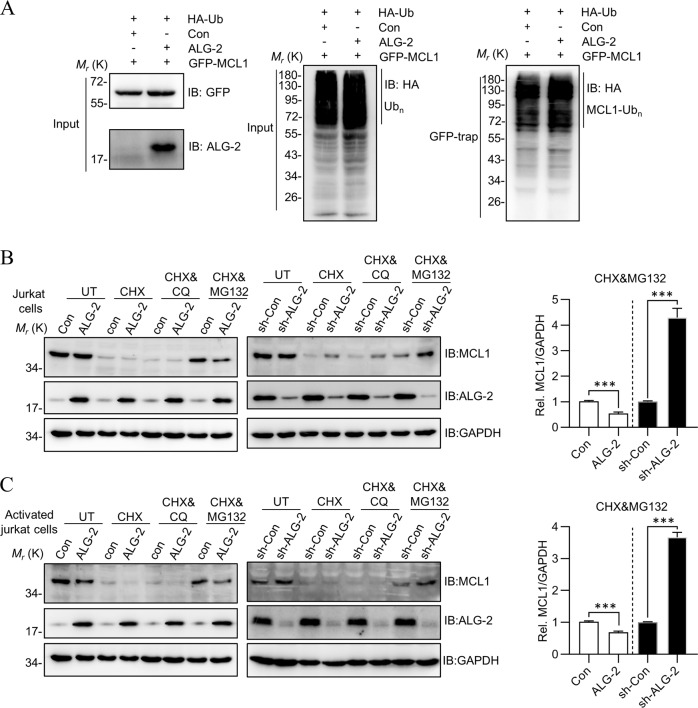
ALG-2 accelerates MCL1 degradation. **a** The effect of ALG-2 on the ubiquitination of MCL1. HEK293T cells were transfected with GFP-MCL1, HA-Ubiquitin, with or without ALG-2 for 48 h and lysed by RIPA buffer. Cell lysates were immunoprecipitated with GFP-Trap sepharose beads and analyzed by HA antibody using western blot. **b**, **c** ALG-2 accelerates MCL1 degradation in Jurkat cells. The ALG-2-OX and ALG-2-KD Jurkat cells (**b**) or activated cells (**c**) were seeded in six-well plates and treated with CHX, CQ or MG132 for 8 h, and lysed in RIPA buffer for detection.

Because ALG-2 enhanced the activity of the proteasome, and MCL1 degradation was carried out through the ubiquitination-mediated proteasome pathway, we hypothesized that ALG-2 may influence the stability of MCL1. First, no significant change was observed to the ubiquitination level of MCL1 following co-expression of ALG-2 (Fig. 4a). Second, CHX was added into the Jurkat cell culture medium to inhibit protein synthesis and induce cell apoptosis (Fig. 4b). Interestingly, without CHX treatment, in inactivated cells, endogenous MCL1 was stable under both ALG-2-OX and ALG-2-KD (Fig. 4b, first two lanes), but this stability was disrupted by PMA/ionomycin-mediated activation (Fig. 4c, first two lanes). Following CHX treatment, MCL1 was found to be degraded so quickly that it was difficult to distinguish their differences. To investigate the role of ALG-2 in MCL1 degradation, appropriate amount of inhibitors was used to slow down the degradation speed. The supply of MG132 along with CHX, but not chloroquine (CQ), inhibited MCL1 degradation, further supported that the proteasome, but not the lysosome pathway, was involved in this process. With CHX&MG132 treatment, compared to control cells, the residual MCL1 was less in ALG-2-OX cells, but more in the ALG-2-KD cells. This was consistent with ALG-2 having a positive influence on proteasome activity. Additionally, this observation was also held true for activated T cells (Fig. 4c), indicating activation, similar to CHX, was also an initiator of MCL1 degradation. Taken together, ALG-2 accelerated MCL1 degradation by enhancing the activity of the proteasome. It is also noted that when cells were under normal growth conditions without chemical treatment (PMA/Ionomycin for activation or CHX for protein synthesis inhibition), ALG-2 had no influence on the levels of MCL1, indicating that ALG-2 did not initiate the degradation of MCL1, but only accelerated this process after the degradation of MCL1 had begun, either due to cell activation or by cellular stress.

### The acceleration of MCL1 degradation by ALG-2 relies on its interaction with Rpn3

**Fig. 5:**
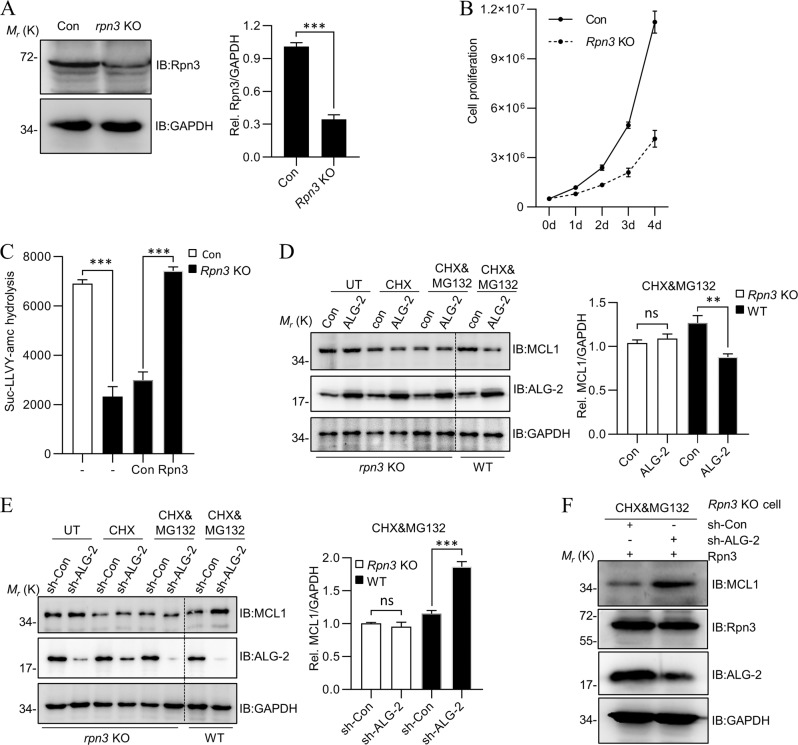
The acceleration of MCL1 degradation by ALG-2 relies on its interaction with Rpn3. **a** The expression level of Rpn3 in Rpn3-KO Jurkat cell line. The protein levels of Rpn3 and GAPDH are determined by western blotting. **b** The proliferation rate of Rpn3-KO Jurkat cells. The assay was started with 500,000 cells and measured by Countstar cell-counter system. Data shown were representative of three independent experiments. **c** The proteasome activity in Rpn3-KO cell line was impaired, but was rescued with re-expression of Rpn3. **d**, **e** ALG-2 loses the ability to accelerate MCL1 degradation in Rpn3-KO Jurkat cell line. Rpn3-KO Jurkat cells were infected with ALG-2 retrovirus (**d**) or sh-ALG-2 retrovirus (**e**) for 40 h, then treated with CHX and MG132 for another 8 h. Cells were collected and lysed in RIPA buffer for detection. **f** The effect of ALG-2 on the stability of MCL1 when Rpn3 was rescued in *Rpn3*-KO cell. Data shown were representative of three independent experiments. Error bars indicate SD. ns, no significance; ***P < 0.001.

To ensure that the effect of ALG-2 on MCL1 worked through Rpn3, a *rpn3* knockout (KO) Jurkat cell line was established. As expected, due to the essential role of Rpn3, we can only obtain a cell line that had a reduced level (Fig. 5a), but not complete loss of Rpn3 (~30% Rpn3 protein remaining), with a slower growth rate compared to WT (Fig. 5b). The protein levels of Rpn6 and Rpn11 were not interfered (Fig S2). As expected, *rpn3* KO cells exhibited lower proteasome activity compared to WT, but the proteasome activity was fully restored when Rpn3 was re-introduced (Fig. 5c). Moreover, as shown in Fig. 5d, e, following CHX or CHX&MG132 treatment, when ALG-2 was further overexpressed or knocked-down in *rpn3* KO cells, the effect of ALG-2 on the stability of MCL1 was diminished. However, the effect of ALG-2 on the stability of MCL1 was restored when Rpn3 was re-introduced in this cell line (Fig. 5f). All these observations supported the notion that ALG-2 affected the stability of MCL1 through a direct interaction with Rpn3.

### ALG-2 enhances T cell apoptosis

**Fig. 6:**
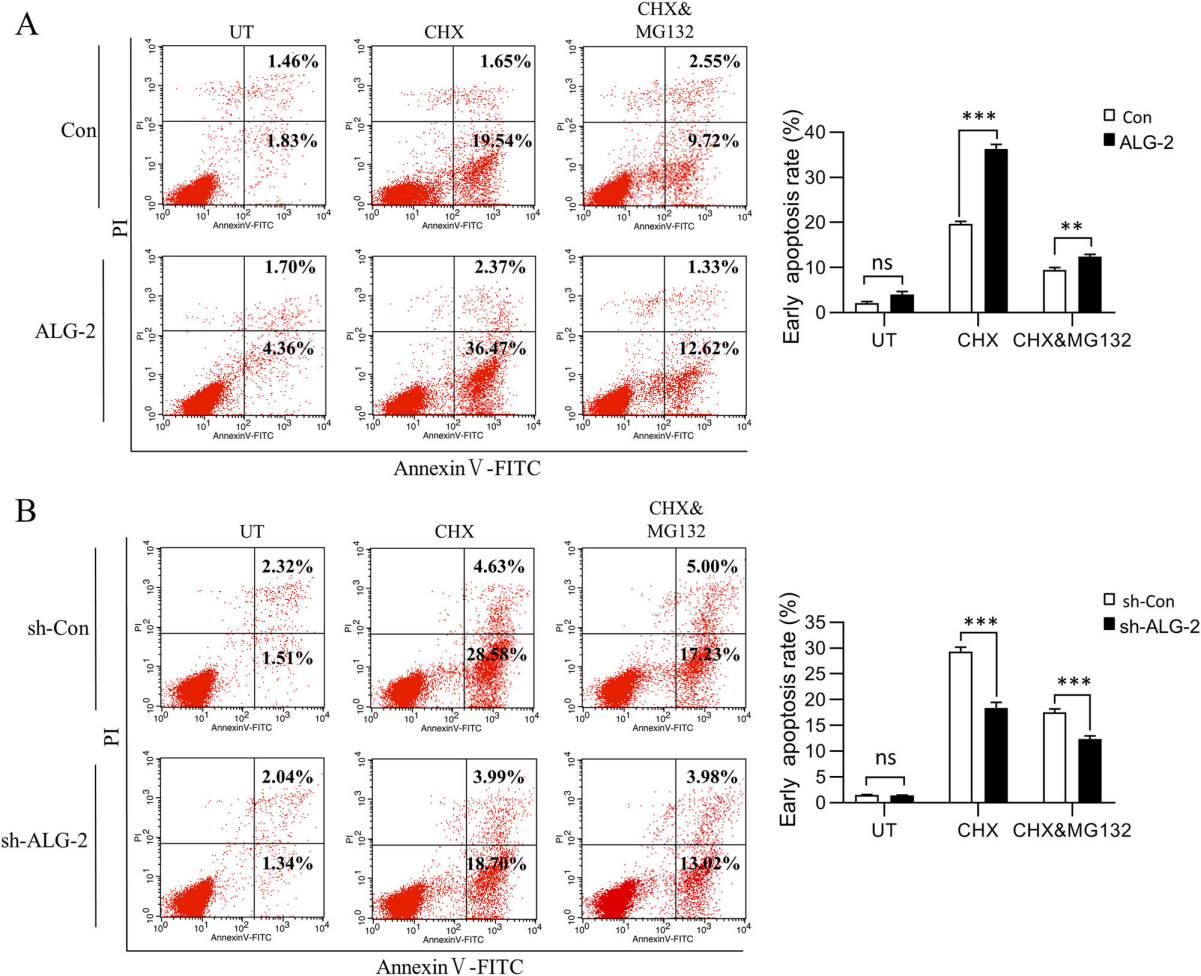
ALG-2 enhances T cell apoptosis. The ALG-2-OX (**a**) and ALG-2-KD (**b**) Jurkat cells were treated with CHX (20 μM) in the presence or absence of MG132 (2 μM) for 5 h, then collected for staining with Annexin V-FITC/PI Apoptosis Detection kit. The apoptotic ratio was measured by FACS Calibur flow cytometer and analyzed by FlowJo software. Data shown were representative of three independent experiments. Error bars indicate SD. **P < 0.01; ***P < 0.001.

Due to ALG-2 accelerating the degradation of MCL1 after the initiation of apoptosis, we speculated that ALG-2 may enhance cell apoptosis. Jurkat cells were counted to establish an apoptotic ratio following CHX treatment by flow cytometry. As expected, overexpression of ALG-2 increased the ratio of apoptotic cells (Fig. 6a), while knockdown decreased it (Fig. 6b). The supply of MG132 rescued the cells from apoptosis partially, indicating that the cells underwent apoptosis via a proteasome-mediated degradation process.

### ALG-2 affects MCL1 stability and cell apoptosis induced by serum starvation

**Fig. 7:**
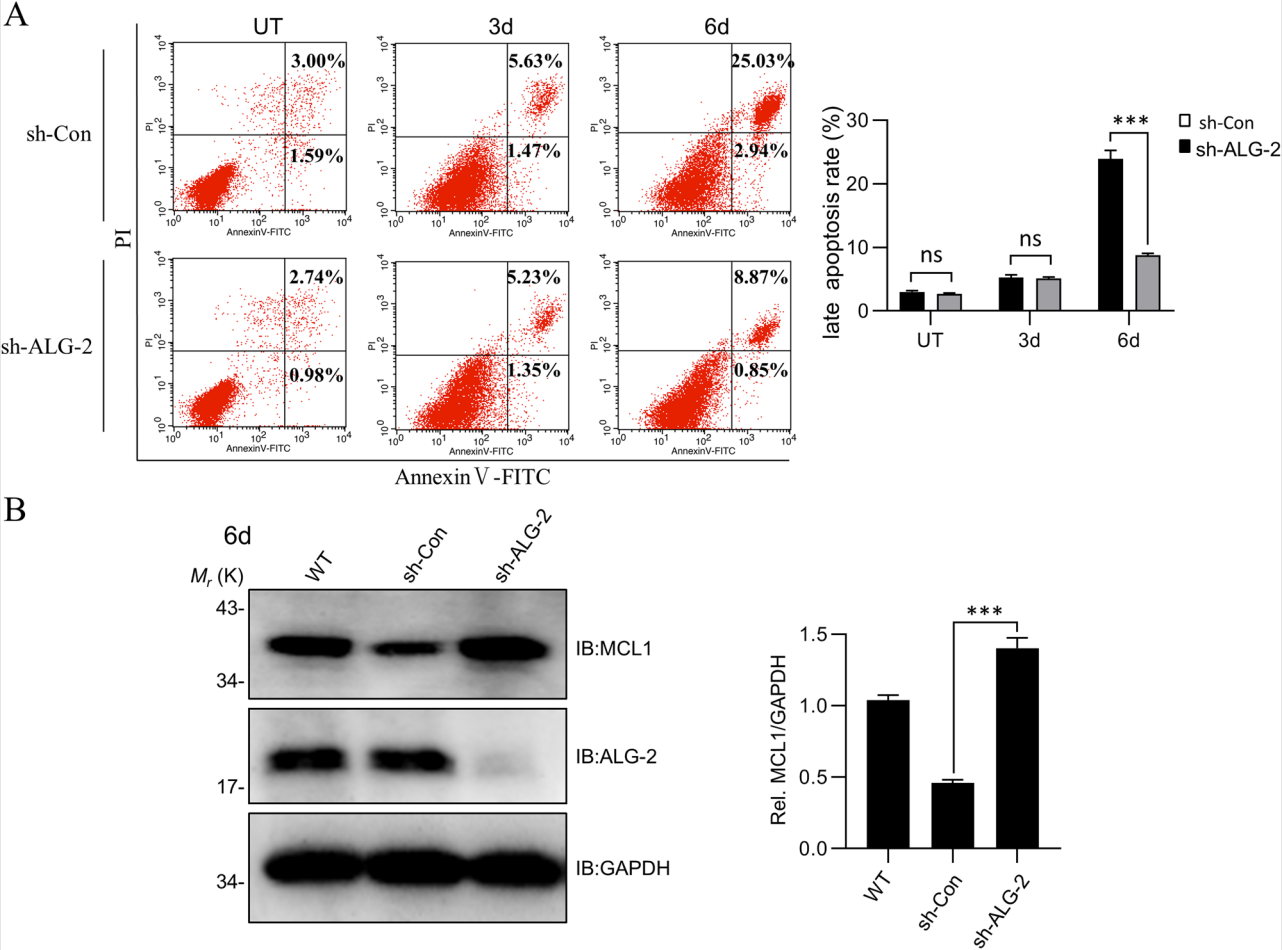
ALG-2 affects MCL1 stability and cell apoptosis induced by serum starvation. **a** ALG-2 knockdown attenuates cell apoptosis induced by serum starvation. The ALG-2-KD Jurkat cells were cultured in 1% FBS medium, and were measured by Annexin V-FITC/PI Apoptosis Detection kit using FACS Calibur flow cytometer in 0d, 3d, and 6d. **b** The changes of MCL1 protein level in ALG-2-KD cells cultured in 1% FBS medium. 1.5 × 10^6^ Jurkat cells were collected on the 6th day and detected by MCL1 antibody. Data shown were representative of three independent experiments. Error bars indicate SD. ns, no significance; ***P < 0.001.

Last, we investigated whether ALG-2 played a role when the cells were exposed to serum starvation, a physiologically relevant condition in the waning phase of activated T cells. Following serum starvation (1% FBS) for six days, compared to WT, ALG-2-KD Jurkat cells showed much less cell apoptosis, mainly in late stage (Fig. 7a), in accordance with more MCL1 within cells (Fig. 7b), further supporting the role of ALG-2 in apoptosis under physiologically relevant conditions.

**Fig. 8:**
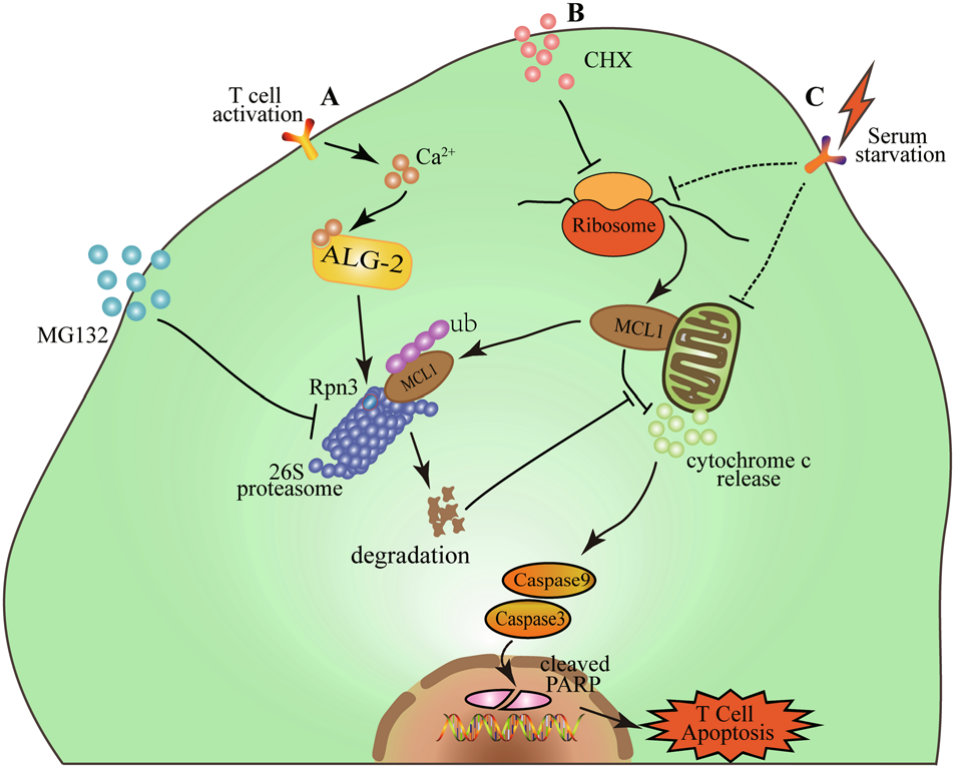
The model of ALG-2 function in T cell apoptosis. Upon T cell activation, the intracellular calcium level increases dramatically. ALG-2 binds to available calcium ion, and enhances the activity of proteasome by interacting with Rpn3. Consequently, the degradation of MCL1 is accelerated, and T cell apoptosis initiated (**a**). MG132 can inhibit the activity of proteasome and decelerate MCL1 degradation, thus rescue cells from apoptosis. Other stimuli, such as CHX and serum starvation, also trigger apoptosis by regulating MCL1 availability, a process affected by ALG-2 (**b**, **c**).

Taken together, our study provided a direct link between T cell activation and activation-induced cell death with the function of ALG-2. A schematic model is shown in Fig. 8.

## Discussion

Several Bcl-2 proteins, in coordination with death receptors, have been suggested to be involved in the apoptotic process during the contraction phase of the immune response. Proapoptotic proteins such as Bim and Puma as well as antiapoptotic proteins Bcl-2, Bcl-xL, A1, and MCL1 have all been proposed in various studies. However, of interest for this study, MCL1 has a number of unique roles. First, MCL1 has been established as one of the key molecules in T cell development and survival, with *Mx-Cre*-mediated ablation of MCL1 leading to the rapid loss of hematopoietic cells^44^. Second, MCL1 is essential for supporting an efficient effector T cell response upon acute viral infection^9^. Third, MCL1 has a very short half-life, with the protein levels regulated promptly upon environmental cues. T cell contraction, following T cell activation, is initiated by the loss of pro-survival cytokines upon antigen clearance. This process may quickly destabilize MCL1, and lead to the loss of T cells and a decrease to the immune response. MCL1 is degraded by the 26S proteasome pathway following ubiquitination by several E3 ligases. The phosphorylation of several serine/threonine residues within the PEST sequence by survival-related kinases, such as JNK and ERK, has also been shown to influence the stability of MCL1. Except the status of MCL1 itself, including modification, localization, and interaction with binding partners, cytosolic factors surrounding MCL1 also have a significant influence on the stability of the protein. MCL1 is degraded by the proteasome, and therefore, unsurprisingly, the proteasome activity has a direct influence on the turnover rate of MCL1. The proteasome activity is also tightly associated with T cell activation. Activated T cells, in response to the increased energy demand, have higher activity of the proteasome. In this study, we verified the tight correlation between the levels of MCL1 and T cell apoptosis, and confirmed that the levels of MCL1 are directly associated with the activity of proteasome. Furthermore, we identified a new regulator of proteasome activity, ALG-2, which had a direct influence on T cell apoptosis through the regulation of proteasome activity, and consequently affecting the stability of MCL1.

Since its identification more than 20 years ago, ALG-2 has attracted a lot of attention regarding its exact roles in T cells, cancer cells, and various other secretory cells. More than a dozen interacting partners of ALG-2 have been identified, including proteins involved in vesicle transportation, secretion, calcium signaling, and RNA processing. ALG-2 was first identified as a proapoptotic factor in T cell hybridoma, but later on studies have argued against its pivotal role in T cell apoptosis, since no abnormity was observed in *alg-2* knockout mice^45^. The T cell development and apoptosis pathway has also been shown to be unaffected by the absence of ALG-2. Despite these discrepancies, the function of ALG-2 is still acknowledged by more and more studies. The role of ALG-2 in vesicle transportation has been proven repeatedly, including its role in the controlling of the ER exit site, the controlling of the size of COPII vesicles, and its critical role in the repair of radiation-induced plasma membrane damage^35,36^. Our previous study also found an important role of ALG-2 in fighting against HIV infection^28^. All these studies warrant a re-investigation of ALG-2 function in T cell apoptosis. In this study, we found that ALG-2 responded to the calcium level upon T cell activation and influenced T cell apoptosis, and thus coupled T cell activation and apoptosis processes together. Our study also established a direct link between ALG-2 and proteasome activity, and thereby echoed the original finding of ALG-2 function in T cells.

The discrepancies in the studies surrounding ALG-2 function in T cell apoptosis need to be scrutinized, and we attributed these discrepancies to several reasons^45^. First, despite a definite role in T cell apoptosis, ALG-2 might not function alone, with other redundant proteins existing, and the autonomous regulation of expression of redundant proteins in knockout mice might conceal the consequence resulting from absence of ALG-2. Second, the variety of experimental conditions used to measure cell apoptosis in different laboratories needs to be taken into consideration. As apoptosis induced by various factors, including serum withdrawal, toxins, chemicals, and protein factors such as Fas, all might have different levels of ALG-2 involvement. Third, the time point that was used to detect the apoptotic signal may also be important, as we found that ALG-2 was critical in a specific phase of apoptosis. ALG-2 can influence the proportion of apoptotic cells during the early stage under CHX treatment (Fig. 6), but has a more significant influence on the population during the later stage in the serum starvation conditions (Fig. 7). This discrepancy may be due to the time point chosen for apoptotic detection, for example, rapid apoptotic induction seen within hours versus a slow apoptotic process within days, under each condition.

The activity of the proteasome is critical in the survival of all cell types, and a variety of regulators of the proteasome, such as ALG-2, have already been identified. ALG-2 can coordinate several calcium ions, and can sense the fluctuation of calcium levels within cells. Additionally, ALG-2 regulates the endoplasmic reticulum (ER)–Golgi vesicular transport, endosomal biogenesis, and membrane repair, as all of these processes involve calcium. T cell activation is also accompanied by a sharp increase of cytosolic calcium. In our study, we found that ALG-2 influenced proteasome activity, and was critical for the increase of the proteasome activity following T cell activation, which was due to its calcium-binding ability. As ALG-2 may be already pre-occupied by calcium ions partially prior to activation, the function of ALG-2 on the activity of the proteasome due to increases in calcium after T cell activation may be under-estimated in our experimental system.

In summary, our study reinvestigated the function of ALG-2 in T cell apoptosis, and echoed the original finding of ALG-2 function in T cell survival. With a novel direct interaction with Rpn3, ALG-2 may regulate the activity of the proteasome in response to intracellular calcium level changes, linking calcium signaling and proteasome degradation. However, since Jurkat T cells have some differences in their apoptotic process compared with that of primary T lymphocytes in living organisms, more studies need to be carried out under more physiological conditions to verify these conclusions further in the future.

## Materials and methods

### Cells, reagents, antibodies, and constructs

HEK293T, HeLa, and Jurkat cells were obtained from ATCC. HEK293T and HeLa cells were cultured in Dulbecco's Modified Eagle's Medium (DMEM; Corning, NY, USA) supplemented with 10% fetal bovine serum (FBS; Clark Bioscience, VA, USA), and Jurkat cells were cultured in Roswell Park Memorial Institute (RPMI)-1640 medium (Corning) supplemented with 10% FBS at 37 °C under a 5% CO_2_ atmosphere. Human primary peripheral blood mononuclear cells (PBMCs) were isolated from peripheral blood buffy coats by density gradient centrifugation. Human peripheral blood samples were collected from three healthy adult donors and obtained from First Central Hospital of Tianjin (Tianjin, China). The samples were collected after approval by the academic committee of Nankai University and signing written informed consent by donors. The peripheral blood was diluted with equal volume PBS-EDTA (containing 0.05 M EDTA), and was added into equal volume human peripheral blood lymphocyte separation medium (Solarbio, Beijing, China) according to the manufacturer’s instructions. PBMCs were cultured in RPMI-1640 medium with 10% FBS, 100 U/ml Penicillin/Streptomycin (Solarbio), 2 mM l-glutamine (Solarbio). Non-viable cells and cell number were examined with trypan blue staining using a Countstar cell-counter system.

Adherent cells were transfected with plasmids using polyethylenimine (PEI; Polysciences, IL, USA) when cells were 60% confluent. Cycloheximide (CHX, 20 μM; InvivoGen, San Diego, CA, USA), MG132 (2 μM; InvivoGen) and chloroquine (CQ, 100 μM; InvivoGen) were added in medium to evaluate the degradation of MCL1 in Jurkat cells. Monoclonal Abs against enhanced GFP (eGFP; sc-390394; Santa Cruz CA, USA), hemagglutinin (HA; H3663; Sigma-Aldrich, St Louis, MO, USA), GAPDH (sc-32233; Santa Cruz), and polyclonal Abs against ALG-2 (A6685; Abclonal, Wuhan, China), Rpn3/PSMD3 (12054-1-AP; Proteintech Group, CHI, USA) and MCL1 (16225-1-AP; Proteintech Group) were purchased from the indicated manufacturers. Horseradish peroxidase (HRP)-conjugated anti-mouse and anti-rabbit IgG antibodies were purchased from Sungene Biotech (Tianjin, China). Annexin V-FITC/PI Apoptosis Detection kit was purchased from UE (US Everbright Inc., Suzhou, China).

The cDNA of Rpn3 was a.pngt from Dr. Jiahuai Han (Xiamen University, Xiamen, China). GST tag was fused to the N-terminus of Rpn3, and the fusion gene was constructed into a retroviral vector pQCXIN. pCMV-MMLV-gag-pol and pVSV-G were.pngts from Dr. Wentao Qiao (Nankai University, China). For GST pull down assay, 6 × His was fused to the N-terminus of Rpn3 and GST tag was fused to the N-terminus of ALG-2 in prokaryotic expression system. For GFP-Trap co-immunoprecipitation and fluorescence colocalization analysis, Rpn3 was constructed into an N-terminally eGFP-tagged eukaryotic expression vector pEGFP-C1. pCMV-3HA-ALG-2, pCI-neo-RFP-ALG-2, retroviral vector pQCXIN-ALG-2 and shRNA for human ALG-2 knockdown were constructed accordingly. shRNA-resistant ALG-2 and ALG-2 mutants (E47D, E114D and D169A) and Ub^G76V^-GFP were generated by site-directed mutagenesis as described previously. pCDNA3.1-HA-ubiquitin was a.pngt from Dr. Min Wei (Nankai University, Tianjin, China).

### Retrovirus production and infection

The VSV-G pseudotyped retrovirus was produced by Moloney murine leukemia virus (MMLV)-based retroviral vector system. HEK293T cells (~4 × 10^6^) were seeded in 100 mm dishes 24 h before transfection. Six micrograms of packaging plasmid pCMV-MLV-gag-pol, 6 µg of retroviral vector, and 3 µg of pVSV-G were transfected into cells with a PEI (pH 7, 1 μg/ml) ratio of 4 μl PEI stock to 1 μg plasmid DNA. The media was changed 6 h after transfection and the virus was harvested at 48 h. The supernatants were filtered through 0.2 μm filters and incubated with lenti-virus concentration reagent (Biomega, San Diego, USA) at 4 °C overnight. The mixture was centrifuged at 3000 rpm for 30 min at 4 °C and the virus-containing pellet would be visible. The pelleted particles were resuspended in 1 mL RPMI-1640 medium and spined at 8000 rpm for 2 min at 4 °C to collect purified virus. Three aliquots were snap frozen in a liquid nitrogen bath and stored at −80 °C.

### Co-immunoprecipitation and western blot

HEK293T cells (~4 × 10^6^) were seeded in 100 mm dishes and transfected with HA-ALG-2 or its mutants (4 µg), eGFP-Rpn3 or control plasmid (4 µg) by PEI method. Cells were harvested 48 h after transfection and lysed using RIPA buffer (50 mM Tris HCl pH 8, 150 mM NaCl, 1% NP-40, 0.1% SDS, 0.5% sodium deoxycholate, 1 mM PMSF) for 1 h on ice. Cell lysates were prepared by sonication and centrifuged at 12,000 g for 10 min at 4 °C. For GFP-Trap co-immunoprecipitation, the supernatants (as input) were incubated overnight at 4 °C with ~40 μL GFP-Trap beads (Chromotek, Germany). For co-immunoprecipitation assay, the supernatants were incubated overnight at 4 °C with ~40 μL protein A-Sefinose^TM^ Resin (BIO Basic Inc., Shanghai, China) and 0.3 μg HA tag antibody. Next, the beads were recovered and washed with RIPA buffer four times before detected by western blot. The lysates were boiled with SDS loading buffer at 100 °C for 10 min, and fractionated by SDS-PAGE. The proteins were transferred onto a PVDF membrane (0.45 μm, GE Healthcare), and blocked using 5% skim milk for 1 h at room temperature. The protein bands were probed with the indicated antibodies and visualized using electro-generated chemiluminescence (ECL) imaging in a Tannon-5500 gel imager.

### GST pull down coupled with LC-MS/MS

Jurkat cells (~5 × 10^5^) were seeded in six-well plates and infected with pQCXIN-GST-Rpn3 or pQCXIN-GST retrovirus. The media was changed 24 h after infection by 800 rpm centrifugation for 90 min and cells were harvested at 72 h. The lysates were prepared by 500 μL RIPA buffer (as described above) for 30 min on ice and then centrifuged at 12,000 g for 10 min at 4 °C. The supernatant was incubated with ~30 μL Glutathione Sepharose beads (GE Healthcare) overnight at 4 °C. The Sepharose beads were washed three times with 1 ml pre-cooled PBS, and the precipitates were boiled with SDS loading buffer at 100 °C for 10 min. The supernatant was analyzed in the Beijing Genomics Institute (BGI, China) for LC-MS/MS.

### His-tag pull down

For His-tag pull-down in prokaryotic expression, His-tagged Rpn3, PKM2, and GST-tagged ALG-2 were expressed in *Escherichia coli* BL21 (DE3) strain (Novagen), and cultured in LB medium with kanamycin (50 μg/ml) and Ampicillin (100 μg/ml), respectively. Recombinant protein expression was induced by isopropyl β-D-1-thiogalactopyranoside (IPTG, 200 μM) at 16 °C for 12 h. The cells were harvested by 4,000 rpm centrifugation and were mixed by re-suspended in binding buffer (50 mM Tris-HCl, 500 mM NaCl, pH 8.0). Cell pellets were lysed by sonication and removed by 18,000 rpm centrifugation at 4 °C. The recombinant proteins were purified by Ni Sepharose beads (GE Healthcare) and washed three times by pre-cooled binding buffer with 20 mM imidazole. The samples were boiled with SDS loading buffer at 100 °C for 10 min and fractionated by SDS-PAGE and detected by coomassie blue staining to confirm the interaction between Rpn3 and ALG-2.

### CRISPR-Cas9

The CRISPR genomic guide RNA sequence (gRNA) to human Rpn3 (PSMD3) was taken from a lentiCRISPR sgRNA library designed by Feng Zhang^46^. The Rpn3 gRNA oligos were annealed in TE buffer as recommended protocol and ligated into lentiCRISPR, a construct possessing an ability to simultaneously deliver a sgRNA, Cas9, and a puromycin selection marker into target cells^46^. Lentivirus was produced similarly to retrovirus except a HIV-based packaging plasmid psPAX2 and concentrated as described above. After 48 h following infection, the cells were selected with 1 μg/ml puromycin for 10 days to eliminate wild type cells. Monoclonal Jurkat cell was obtained by limited dilution method and expanded in 96-well plates. Clonal Jurkat knockout cells were characterized for a reduced Rpn3 protein expression. Rpn3 gRNA targeting sequence: 5ʹ-ACGCTTCGGCATGACGCAGA-3ʹ.

### Flow cytometry

Jurkat cell lines (~1 × 10^6^) were seeded in six-well plates and treated with CHX (20 μM) in the presence or absence of MG132 (2 μM) for 5 h in 37 °C incubator. The Jurkat cells were collected and stained with Annexin V-FITC/PI Apoptosis Detection kit according to the manufacturer’s instructions. The data were collected using a FACS Calibur flow cytometer (BD Biosciences, Franklin Lakes, NJ, USA) and analyzed to quantify the apoptotic cells using FlowJo software.

### Fluorescence co-localization and GFP fluorescence intensity

HeLa cells (~1 × 10^5^) were seeded onto cell slides in 12-well plate and transfected with GFP-Rpn3 and RFP-ALG-2. Cells were fixed with 4% paraformaldehyde for 15 min at room temperature and permeabilized with 4% paraformaldehyde plus 0.1% Triton X-100 for 10 min. Then cells were stained with DAPI in mounting medium and analyzed with a Leica TCS SP5 confocal laser scanning microscope. HeLa cells were transfected with ALG-2, GFP or Ub^G76V^-GFP, and treated with CHX and in the absence or presence of MG132. Jurkat cell lines were infected with VSV-G pseudotyped retrovirus GFP or Ub^G76V^-GFP for 36 h, and were activated by treated with PMA (50 ng/ml) and ionomycin (1 µM) for 2 h, and then continually cultured for 6 h with CHX and in the absence or presence of MG132. The GFP fluorescence was analyzed with a Zeiss MF53 inverted fluorescence microscope.

### 26S proteasome activity assay

Jurkat cell lines were activated by treated with PMA (50 ng/ml) and ionomycin (1 µM) for 10 h and subsequently cultured in fresh medium for another 12 h. The Jurkat cells or activated cells (~2 × 10^6^) were collected and incubated for 1 h on ice in digitonin permeability buffer, which consists of 50 mM HEPES, pH 7.5, 5 mM MgCl_2_, 1 mM DTT, 0.5 mM EDTA, 250 mM sucrose, 2 mM ATP, and 0.025% digitonin. Cytosolic extracts were generated by slight sonication and then “squeezed out” by centrifugation at 20,000 g at 4 °C for 20 min. Cytosol was incubated for 1 h at 37 °C in reaction buffer containing 50 mM Tris–HCl (pH 7.5), 40 mM KCl, 5 mM MgCl_2_, 0.5 mM ATP, 1 mM DTT, 0.2% BSA, and 100 μM fluorogenic peptide Suc-LLVY-AMC^47^. AMC fragment cleavage was quantified by a microtiter plate reader (Cytation 5, BioTek Instruments, Vermont, USA) at *λ*^EX^ = 380 nm/*λ*^EM^ = 460 nm.

### Statistical analysis

All data in histograms were displayed as the mean ± SD. Statistical analysis was performed by using GraphPad Prism software (version 8.0; Inc., La Jolla, CA, USA). Significant difference was calculated using unpaired Student's *t*-test. *P* < 0.05 was considered to be statistically significant.

## Supplementary information

Supplementary figure legends

Peptides identified in the LC-MS/MS study (GST-Rpn3 VS. GST)

ALG-2 enhances the activity of proteasome, detected with Ub^G76V^-GFP

The protein level of Rpn6 and Rpn11 were not changed in Rpn3 knockout cell line
