# Supplementary material for: ALG-2 couples T cell activation and apoptosis by regulating proteasome activity and influencing MCL1 stability
Source: Cell Death Dis. 2020 Jan 2;11(1):5. doi: 10.1038/s41419-019-2199-4 (PMC6952393; doi:10.1038/s41419-019-2199-4)
Supplement: Supplementary file 1 — Supplementary figure legends [file 41419_2019_2199_MOESM1_ESM.docx]

**Supplementary figure legends**

**Fig. S1** ALG-2 enhances the activity of proteasome, detected with Ub^G76V^-GFP.

(A) ALG-2 promotes the degradation of Ub^G76V^-GFP in HeLa cells. HeLa cells were transfected with HA-ALG-2, GFP or Ub^G76V^-GFP, cultured for 36 h, then cultured for another 6 h in the presence of 20 μM CHX, with or without 5 μM MG132. Ub^G76V^-GFP degradation was measured with fluorescence intensity. (B-C) ALG-2 promotes the degradation of Ub^G76V^-GFP in activated Jurkat cells. The ALG-2-OX and ALG-2-KD Jurkat cells were infected with GFP or Ub^G76V^-GFP retrovirus for 48 h, then activated with PMA (50 ng/ml) and ionomycin (1 µM) for 10 h. Fluorescence intensity was quantitated on the right. Scale bars: 100 μm. Error bars indicate SD. ns, no significance; ***, P <0.001.

**Fig. S2** The protein level of Rpn6 and Rpn11 were not changed in Rpn3 knockout cell line.

Rpn3 knockout cells or control Jurkat cells (1.5×10^6^) were collected and lysed by RIPA buffer. The protein levels of Rpn6, Rpn11 and GAPDH are determined by western blotting.
