# Supplementary material for: ALG-2 couples T cell activation and apoptosis by regulating proteasome activity and influencing MCL1 stability
Source: Cell Death Dis. 2020 Jan 2;11(1):5. doi: 10.1038/s41419-019-2199-4 (PMC6952393; doi:10.1038/s41419-019-2199-4)
Supplement: Supplementary file 2 — Peptides identified in the LC-MS/MS study (GST-Rpn3 VS. GST) [file 41419_2019_2199_MOESM2_ESM.pdf]

Table S1 Peptides identified in the LC-MS/MS study (GST-Rpn3 VS. GST)

prot_acc	prot_desc	prot_mass	pep_score	pep_expect	pep_seq
O43242	26S proteasome non-ATPase regulatory subunit 3 GN=PSMD3	61054	139.82	1.80E-13	SVFPEQANNNEWAR
P68363	Tubulin alpha-1B chain GN=TUBA1B	50804	137.35	1.00E-13	TIGGGDDSFNTFFSETGAGK
O43242	26S proteasome non-ATPase regulatory subunit 3 GN=PSMD3	61054	99.4	1.9E-09	AIQLEYSEAR
P0DMV8	Heat shock 70 kDa protein 1A GN=HSPA1A	70294	94.68	5.10E-09	IINEPTAAAIAYGLDR
P68032	Actin, alpha cardiac muscle 1 GN=ACTC1	42334	93.88	7.10E-09	SYELPDGQVITIGNER
Q562R1	Beta-actin-like protein 2 GN=ACTBL2	42318	93.88	7.10E-09	SYELPDGQVITIGNER
P10809	60 kDa heat shock protein, mitochondrial GN=HSPD1	61187	93.34	1.40E-09	TALLDAAGVASLLTTAEVVVTEIPK
P68371	Tubulin beta-4B chain GN=TUBB4B	50255	89.07	2.00E-08	GHYTEGAELVDSVLDVVR
P61019	Ras-related protein Rab-2A GN=RAB2A	23702	78.09	2.80E-07	TASNVEEAFINTAK
Q9H0D6	5~-3~ exoribonuclease 2 GN=XRN2	109426	75.45	4.20E-07	AALEEVYPDLTPEETR
**O75340**	**Programmed cell death protein 6 GN=PDCD6**	**21912**	**72.37**	**6.90E-07**	**DNSGMIDKNELK**
P62979	Ubiquitin-40S ribosomal protein S27a GN=RPS27A	18296	70.44	7.90E-07	ESTLHLVLR
**O75340**	**Programmed cell death protein 6 GN=PDCD6**	**21912**	**66.67**	**1.60E-06**	**SGVISDTELQQALSNGTWTPFNPVTVR**
P46439	Glutathione S-transferase Mu 5 GN=GSTM5	25829	62.54	8.20E-06	ITQSNAILR
Q96DA2	Ras-related protein Rab-39B GN=RAB39B	24835	54.91	5.90E-05	LQIWDTAGQER
P62847	40S ribosomal protein S24 GN=RPS24	15413	51.4	0.00011	TTPDVIFVFGFR
O00487	26S proteasome non-ATPase regulatory subunit 14 GN=PSMD14	34726	47.87	5.70E-05	AVAVVVDPIQSVK
Q16643	Drebrin GN=DBN1 PE=1 SV=4	71842	47.09	0.00021	LELLAAYEEVIR
Q9NVI7	ATPase family AAA domain-containing protein 3A GN=ATAD3A	71610	46.29	0.0003	ITVLEALR
Q9Y399	28S ribosomal protein S2, mitochondrial GN=MRPS2	33513	43.54	0.00049	LLFGPTVR
Q9BWT3	Poly(A) polymerase gamma GN=PAPOLG	83093	42.94	0.00092	LRSLDIR
Q93008	Probable ubiquitin carboxyl-terminal hydrolase FAF-X GN=USP9X	295515	39.72	0.0018	LAQQISDEASR
Q6Q0C0	E3 ubiquitin-protein ligase TRAF7 GN=TRAF7	76386	37.88	0.0049	IWDIR
Q16186	Proteasomal ubiquitin receptor ADRM1 GN=ADRM1	42412	34.04	0.0043	YLVEFR
P17980	26S protease regulatory subunit 6A GN=PSMC3	49458	32.68	0.0031	VDILDPALLR
Q96J02	E3 ubiquitin-protein ligase Itchy homolog GN=ITCH	103593	32.09	0.0068	IYYVDHFTR
Q96L21	60S ribosomal protein L10-like GN=RPL10L	24959	30.72	0.014	IFDLGR
P62191	26S protease regulatory subunit 4 GN=PSMC1	49325	30.16	0.0039	IETLDPALIRPGR
P35998	26S protease regulatory subunit 7 GN=PSMC2	49002	28.05	0.017	FELLAR
O00308	NEDD4-like E3 ubiquitin-protein ligase WWP2 GN=WWP2	99420	26.76	0.013	NYEQWQSQR
Q5XPI4	E3 ubiquitin-protein ligase RNF123 GN=RNF123	149960	23.68	0.014	LTIAILR
O95714	E3 ubiquitin-protein ligase HERC2 GN=HERC2	533510	20.93	0.034	VVVDLLK
P0C646	Olfactory receptor 52Z1 GN=OR52Z1 PE=3 SV=1	33827	20.67	0.035	IGIAAVVR
Q96DA2	Ras-related protein Rab-39B GN=RAB39B	24835	6.49	4	LAAAYGMKYIETSAR
Q16186	Proteasomal ubiquitin receptor ADRM1 GN=ADRM1	42412	5.29	5.1	AGKMSLK
O95714	E3 ubiquitin-protein ligase HERC2 GN=HERC2	533510	3.55	4.2	KSSRPAGKGVEGLAR
