# Supplementary figures and images for: ALG-2 couples T cell activation and apoptosis by regulating proteasome activity and influencing MCL1 stability
Source: Cell Death Dis. 2020 Jan 2;11(1):5. doi: 10.1038/s41419-019-2199-4 (PMC6952393; doi:10.1038/s41419-019-2199-4)

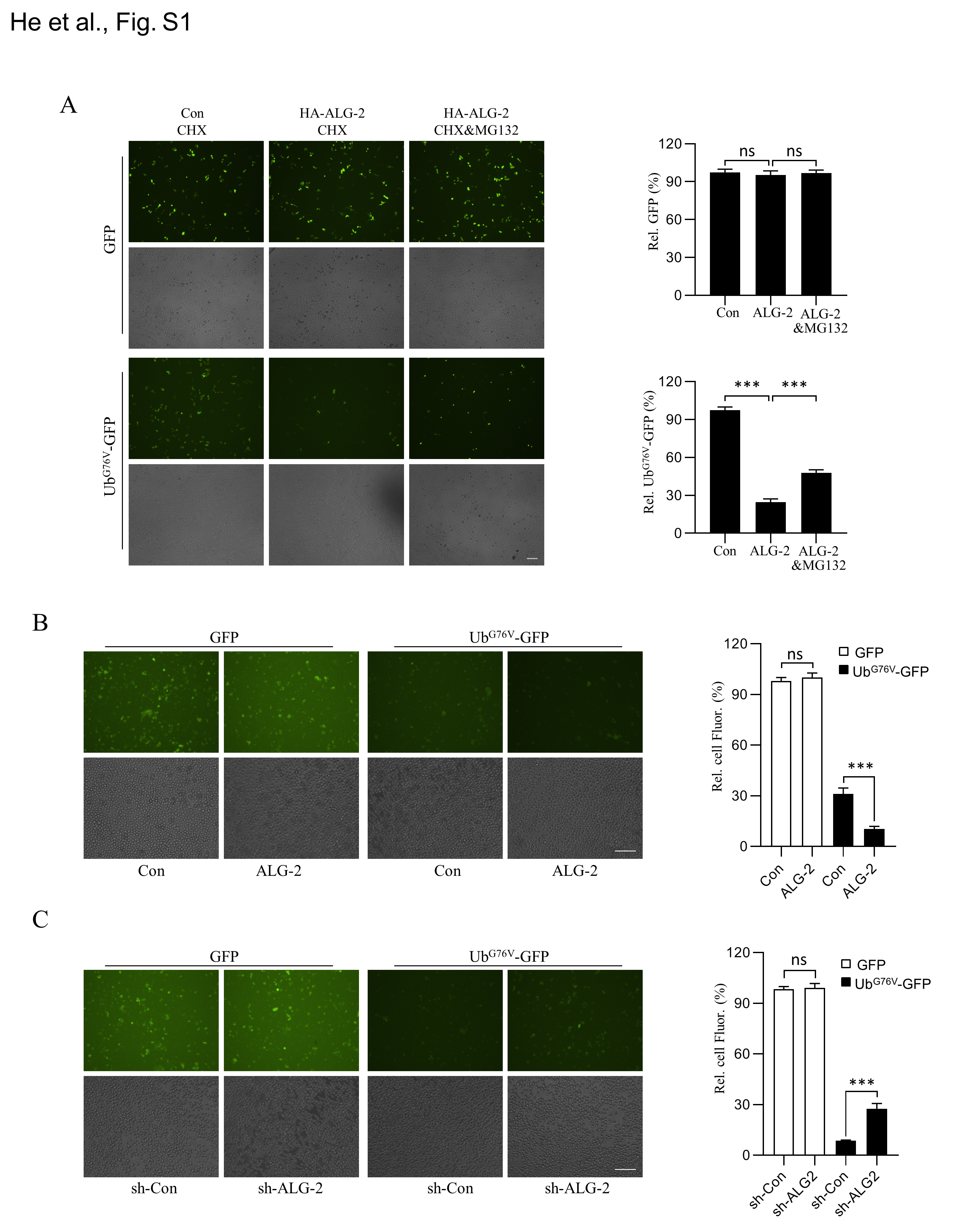

Supplement: Supplementary file 3 — ALG-2 enhances the activity of proteasome, detected with UbG76V-GFP [file 41419_2019_2199_MOESM3_ESM.tif]

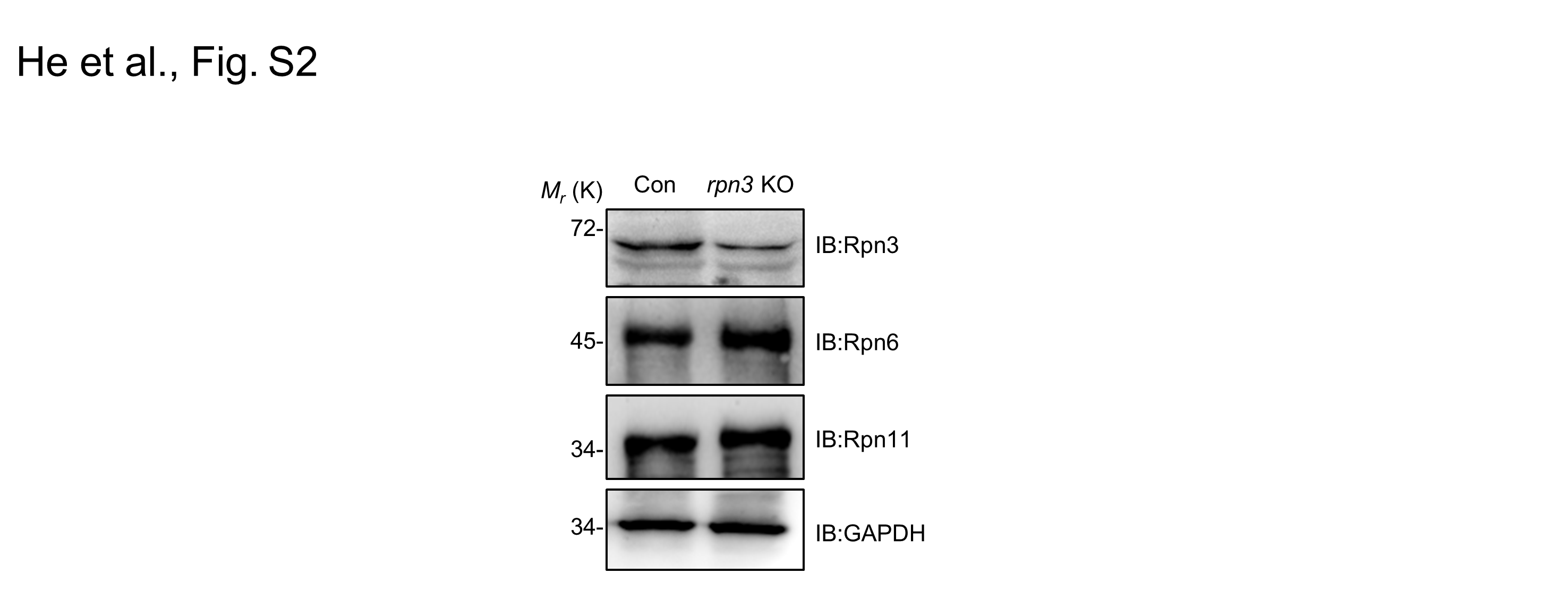

Supplement: Supplementary file 4 — The protein level of Rpn6 and Rpn11 were not changed in Rpn3 knockout cell line [file 41419_2019_2199_MOESM4_ESM.tif]
